# Physiological and Biochemical Changes in Sugar Beet Seedlings to Confer Stress Adaptability under Drought Condition

**DOI:** 10.3390/plants9111511

**Published:** 2020-11-07

**Authors:** Md. Jahirul Islam, Ji Woong Kim, Mst. Kohinoor Begum, Md. Abu Taher Sohel, Young-Seok Lim

**Affiliations:** 1 Department of Bio-Health Convergence, Kangwon National University, Chuncheon 24341, Korea; jahirulislam213@gmail.com (M.J.I.); jiwoongki@naver.com (J.W.K.); 2 Physiology and Sugar Chemistry Division, Bangladesh Sugarcrop Research Institute, Ishurdi 6620, Pabna, Bangladesh; kohinoorbegum.bsri@gmail.com; 3 Agronomy and Farming System Division, Bangladesh Sugarcrop Research Institute, Ishurdi 6620, Pabna, Bangladesh; atsohel@yahoo.com

**Keywords:** sugar beet cultivars, drought, osmolytes, antioxidant enzymes, secondary metabolites, and antioxidant capacity

## Abstract

The present study was conducted to examine the adaptability of 11 sugar beet cultivars grown under drought stress in the controlled glasshouse. The treatment was initiated on 30-day-old sugar beet plants where drought stress was made withholding water supply for consecutive 10 days while control was done with providing water as per requirement. It was observed that drought stress expressively reduced plant growth, photosynthetic pigments, and photosynthetic quantum yield in all the cultivars but comparative better results were observed in S1 (MAXIMELLA), S2 (HELENIKA), S6 (RECODDINA), S8 (SV2347), and S11 (BSRI Sugarbeet 2) cultivars. Besides, osmolytes like proline, glycine betaine, total soluble carbohydrate, total soluble sugar, total polyphenol, total flavonoid, and DPPH free radical scavenging activity were remarkably increased under drought condition in MAXIMELLA, HELENIKA, TERRANOVA, GREGOIA, SV2348, and BSRI Sugar beet 2 cultivars. In contrast, activities of enzymes like superoxide dismutase (SOD), catalase (CAT), and peroxidase (POD) were significantly decreased in all, while the cultivars SV2347, BSRI Sugar beet 1 and BSRI Sugar beet 2 were found with increased ascorbate peroxidase (APX) activity under drought condition. In parallel, polyphenol oxidase (PPO) was increased in all cultivars except HELENIKA. Overall, the cultivars HELENIKA, RECODDINA, GREGOIA, SV2347, SV2348, BSRI Sugar beet 1, and BSRI Sugar beet 2 were found best fitted to the given drought condition. These findings would help further for the improvement of stress adaptive sugar beet cultivars development in the breeding program for drought-prone regions.

## 1. Introduction

To overcome the threats on the agriculture and ecosystem, top-most priority has been given to evaluate the plant response under hostile environments, such as heat, drought, cold, toxic metals, and nutrient deficiency [1,2]. These harsh conditions are collectively referred to as abiotic stress which directly interlinked with the plant growth, development, and overall crop productivity [3,4]. Amidst various detrimental factors, drought is the most repulsive one and extensively impinges on crop growth, yield, and production. The yield and quality of the crop greatly depend on environmental conditions including soil moisture and rainfall pattern [5]. It was estimated that drought caused a potential yield reduction of sugar beet in Europe, ranging from 5 to 30% each year [6]. This event is more tremendous in other regions including arid and semi-arid [7], particularly, where rainfall is relatively low. One such region is South Korea, where drought is a common phenomenon due to the short summer monsoon season [8]. It has observed a prolonged drought from 2013 to 2015 where annual precipitation was below 35–50% of normal levels [9]. United Nations also has classified South Korea as one of the water-deficient countries in the world for its water scarcity problems [10].

Drought tolerant or resurrection plants are quite rare in nature due to the complexity of the adaptation process for the plant. It down-regulates cell enlargement (near about four times higher than cell division) [11] as well as impacts on cell growth by affecting different physiological and biochemical activities [12,13]. However, to enhance the adaptability of a plant when it comes to water scarcity, it is necessary to investigate the sensitivity, morpho-physiological, and biochemical response of a plant under drought stress.

Different morphological traits like relative water content (RWC), chlorophyll fluorescence, grain yield, plant biomass, and plant height have been used to evaluate drought stress [14,15,16,17]. Also, various physiological, morphological, and biochemical responses of plants to water stress have been well documented [18]. For example, growing and water supply condition affects the chlorophyll content of leaves and Fv/Fm which influence the photosynthesis of the varieties [19,20]. Water stress can bring in the change in concentration, composition, and distribution of both primary and secondary metabolites in plant cells which boost their survival capacity against abiotic and biotic stress by following a complex interaction among them [21]. During drought, the plant is subjected to oxidative stress resulting in a series of physiological and biochemical changes that may cause serious metabolic disorders [22]. In response to oxidative damage, plant accumulates different compatible solutes or osmolytes, like proline, sorbitol, phenol, ascorbic acid, glycine betaine, sugars, and polyamines to cope with water stress by maintaining their water relations [23,24]. A previous study showed significantly higher carbohydrates accumulation under severe drought conditions which were considered as stress indicators [25]. However, the response of metabolites to drought stress may be varied among the plant parts and even in genotypes [26]. Therefore, a detailed study on osmoprotectants and their variation of role in different plants to osmotic stress should be analyzed.

Plants under the abiotic stress produce a pool of reactive oxygen species (ROS) which causes an imbalance of component quantities and dysfunction of their normal defensive system [27]. It is stated that the production of reactive molecules and ROS in plants may be stimulated due to the water loss below 70% of RWC [28]. The over-production of such ROS consists of both non-radical (H_2_O_2_) and free radical (O_2_^−^^•^, OH^•^, OH_2_^•^) species, which are persistently detrimental to plants cells [29]. Several previous studies showed a positive relationship between water deficiency and ROS accumulation in *Lotus japonicus* [30] and *Arabidopsis thaliana* [31]. Other studies also mentioned an excessive ROS accumulation in young senescing leaf cells under abiotic stress, which are subject to abolish by a complex mechanism in enzymatic and non-enzymatic pathways [32]. Higher plants also follow a similar mechanism among antioxidant enzymatic and non-enzymatic pathways to protect their cellular and subcellular systems [33]. In general, elevated activity of antioxidant enzymes including ascorbate peroxidase (APX), superoxide dismutase (SOD), catalase (CAT), and peroxidase (POD) is required to quench the intense flux of ROS [34,35]. Therefore, ROS homeostasis by enzymatic and non-enzymatic antioxidants may be an important tool to evaluate plant species to be selected under osmotic stress.

Secondary metabolites are natural compounds produced by the plant and can perform several physiological roles [36]. Secondary metabolites produced by plant cells have not only a significant role in their growth and development process under normal conditions, but also their production under stress can be regulated and used as defensive and tolerance mechanisms [21,37,38]. Also, changing environment can bring in significant change in metabolism and yield [39]. There is little information on sugar beet regarding the effect of drought on secondary metabolites, and their role in stress acclimatization is also unclear. Along with significantly increasing antioxidant activity in plant tissues, secondary metabolites (like polyphenol, flavonoids, and callose) may take part in a defensive role against both biotic and abiotic stress [40,41,42]. Therefore, evaluation of sugar beet seedlings based on antioxidant activity may play a pivotal role in selecting drought-tolerant germplasms for future breeding.

Generally, sugar beet is a biennial crop of temperate regions that requires proper vernalization followed by growing in a long day length (>14 h) for bolting [43,44,45]. The Republic of Korea lies in the temperate zone and geographically it is located in the middle latitudes of the Northern Hemisphere which last 30 years of the average temperature of winter was 0.1–1.1 °C [46]. The average day length of spring (March to May) ranges from 12–14 h and summer (June–August) more than 14 h [47]. So, the climatic position may help to introduce sugar beet breeding in Korea. Moreover, there is no research on sugar beet relates to drought stress was found in Korea as far as our concern. Therefore, the objective of this study was to evaluate sugar beet cultivars against drought stress based on their physiological and biochemical responses for future breeding purposes. 

## 2. Materials and Methods

### 2.1. Experimental Design and Treatment

Sugar beet seedlings were grown in a semi-controlled greenhouse in the department of Bio-Health Convergence, Kangwon National University, Chuncheon, Korea. The environmental condition such as temperature, relative humidity (RH) and average photoperiod was recorded at 30/25 °C (day/night), 60–70% RH and 12 h respectively. Seeds of sugar beet (*Beta vulgaris* L. subs. vulgaris) were provided by Asia seed co., ltd, Seoul, Korea and Bangladesh Sugarcrop Research Institute (BSRI), Ishurdi-6620, Pabna, Bangladesh. The collected 11 genotypes (Table 1) were sterilized [70% (*v/v*) ethanol, 0.1% (*w*/*v*) HgCl_2_ and 0.2% (*w*/*v*) thiram] and placed in the 16 cells hole growing pot (27 × 27 × 6 cm) containing commercial horticultural soil (Bio-soil No. 1, Heungnong Agricultural Materials Mart, Pyeongtaek, Korea). The seedlings were irrigated daily using tap water to the field capacity level up to 30 days. Two treatments were imposed on morphologically uniformed and healthy seedlings, i.e., (i) seedlings were grown under normal growth condition with regular watering and (ii) seedlings were subjected to water deficit by withholding the water supply for 10 days. The sample (youngest completely formed leaves) was collected from each cultivar and immerged immediately in liquid nitrogen followed by freeze-drying and subjected to further analysis.

### 2.2. Determination of Plant Height, Fresh Weight, and Dry Weight 

On the 10th day on the onset of drought treatment, five seedlings from each pot were randomly selected and measured the plant height and fresh weight. Afterward, plants were placed in an oven at 60 °C for 48 h, to determine the dry weight.

### 2.3. Photosynthetic Pigments Analysis

#### 2.3.1. Chlorophyll (Chl) and Carotenoid (Car) Determination

For the determination of photosynthetic pigments, the freeze-dried (50 mg) leaves were extracted (10 mL of 80% acetone) and placed at room temperature for 15 min. The collected extract was transferred into a tube and centrifuged at 4000 rpm for 10 min. The absorbance was taken at 647, 663 and 470 nm, respectively using a spectrophotometer (UV-1800 240 V, Shimadzu Corporation, Kyoto, Japan). Chlorophyll a, Chlorophyll b, Total chlorophyll and Carotenoid were determined according to the formula proposed by Lichtenthaler (1987) [48] and expressed as mg g^−1^ DW:

Chl a = 12.25 × A_663_ − 2.79 × A_647_

Chl b = 21.50 × A_647_ − 5.10 × A_663_

TCh = 7.15 × A_663_ + 18.71 × A_647_

$$Car=\frac{1000\;\times \;A470-1.82\;\times \;\mathrm{Chl}\;a-85.02\;\times \;\mathrm{Chl}\;b\;}{198}$$

#### 2.3.2. Determination of Photosystem II Quantum Yield

The photosynthetic quantum yield (Fv/Fm) of photosystem II (PSII) was measured using the Fluor Pen FP 100 (Photon system Instruments, Czech Republic) by measuring OJIP transient under the dark-adapted condition at least for 20 min [49].

### 2.4. Physiological Traits

#### 2.4.1. Leaf Relative Water Content

Leaves from three seedlings of each genotype from control and water stress treatment were collected and immediately weighted (FW). Later, the leaves were put in deionized water and weighed (g) again after 48 h to determine the leaf turgid weight (TW). Finally, dry weight (DW) was determined by placing in an oven at 60 °C for 48 h. The relative water content (%) of the leaves was calculated according to the following formula [50]:

RWC (%) = [(FW − DW)/(TW − DW)] × 100

#### 2.4.2. Drought Tolerance Index

The drought tolerance index given by Fernandez [51] and adjusted for sugar beet by Ober et al., 2004 [52] was calculated according to the following formula:

DTI=(PDMd/PDMw)/(PDMTd/PDMTw) 

where PDMd is the plant dry matter under drought stress, PDMw is the plant dry matter of the control plants for each genotype and PDMTd and PDMTw are the average values of the plant dry matter for all genotypes under drought stress and control condition, respectively. 

#### 2.4.3. The Membrane Stability Index (MSI)

The membrane stability index (MSI) was taken by the methodology developed by Sairam et al. (2002) [53]. Leaf samples (0.1 g each) were cut into a uniform size (disk shape) and placed into the test tubes with double-distilled water (10 mL) in two sets. One set was kept in a hot water bath (40 °C for 30 min) and its conductivity was recorded (C_1_) using a conductivity meter (HI 5321, HANNA Instruments Inc, Woonsocket, Rhode Island, USA). The second set was also placed in hot water bath (100 °C for 15 min) to record its conductivity (C_2_) again. The membrane stability index (MSI) was measured as: MSI = [1 − (C_1_/ C_2_)] × 100.

### 2.5. Determination of Lipid Peroxidation

Lipid peroxidation was determined by estimating the malondialdehyde (MDA) content in leaves of sugar beet seedlings. MDA was calculated by following the method of Heath and Packer (1968) [54] with slight modification. The freeze-dried leaf sample (25 mg) was macerated in 0.1% trichloroacetic acid (5 mL). The homogenate was centrifuged at 10,000× *g* for 5 min. After that, 4 mL of Trichloroacetic acid (TCA; 20%) containing thiobarbituric acid (0.5%) was added to 1 mL aliquot of supernatant in a test tube. The test tube was placed in a water bath and warmed at 95 °C for 30 min followed by cooled quickly on an ice bath. The resulting mixture was centrifuged again at 10,000× *g* for 15 min and the absorbance was taken at 532 nm. To avoid unspecific turbidity absorbance at 600 nm was subtracted from 532 nm and an extinction coefficient of 155 mM^−1^∙cm^−1^ was used to calculate the concentration. 

### 2.6. Estimation of Hydrogen Peroxide (H_2_O_2_)

The estimation was done according to Singh et al. (2006) [55]. Freeze-dried leaves (25 mg) were extracted in an ice bath with 5 mL of 0.1% (*w*/*v*) TCA and centrifuged at 12,000× *g* for 15 min. For every 0.5 mL of the aliquot of the supernatant, 0.5 mL of 10 mM potassium phosphate buffer (pH 7.0) and 1 mL of 1 M KI were added followed by incubation in darkness for 1 h. The absorbance was measured at 390 nm where 0.1% TCA was used as blank prove in place of leaf extract. A standard H_2_O_2_ curve was prepared to calculate the concentration of H_2_O_2_ in the sample.

### 2.7. Determination of Osmolytes

#### 2.7.1. Free Proline Content

Proline concentration was determined by following the method of Bates et al. (1973) [56]. Approximately 25 mg of freeze-dried plant material was homogenized in 10 mL sulfosalicylic acid (3%) and filtered through Whatman’s filter paper. Two milliliters of the filtrate was mixed with a similar amount (2 mL) of acid-ninhydrin and glacial acetic acid. The mixture was heated for 1 h at 100 °C and cooled immediately on an ice bath. The reaction mixture was extracted with toluene (4 mL). The upper chromophore layer (upper layer) was aspirated and cooled to room temperature. The absorbance was taken at 520 nm with a UV-Vis spectrophotometer (UV-1800 240 V, Shimadzu Corporation, Kyoto, Japan) and calculations were done by using an appropriate proline standard curve.

#### 2.7.2. Glycine Betaine Content

A freeze-dried sample (25 mg) was extracted with methanol (80%) and sonicated for 2 h at room temperature. The solution was filtered through a syringe filter (0.45 µM, Millipore, Bedford, MA, USA). The stock solution of betaine was prepared in ultrapure water (10 mg/mL) and went under serial dilution with the mobile phase to prepare 10, 8, 6, 4, 2, and 1 mg∙mL^−1^, respectively. An improved HPLC method developed by Xu et al. (2018) [57] was used to determined GB in plant leaves. The HPLC system (CBM 20A, Shimadzu Co, Ltd., Kyoto, Japan) with diode array detector (DAD) and a 5 µm C18 column (25 cm × 4.6 mm) were used. The mobile phase consisted of water and acetonitrile 10:90 (*v/v*) mixed with 0.2% phosphoric acid. Sample (2 µL) was injected with a flow rate of 0.5 mL∙min^−1^ where oven temperature was 35 °C and the detection wavelength was 195 nm.

#### 2.7.3. Total Soluble Carbohydrate (TSC) and Sucrose Content

Each sample (25 mg, freeze-dried) was homogenized in 5 mL of ethanol (95%). The insoluble fraction of the extracts were washed with 5 mL of ethanol (70%) followed by centrifuging at 3500 rpm for 10 min and the supernatant was kept in a refrigerator (4 °C) for the determination of TSC and Sucrose content.

TSC content was determined according to Khoyerdi et al. (2016) [58]. Briefly, 0.1 mL of the aliquot was mixed with 1 mL anthrone (200 mg anthrone mixed with 100 mL of 72% sulfuric acid). The mixture was heated at 100 °C for 10 min and then cooled. Total soluble carbohydrate was estimated by using a standard curve, the detection wavelength was 625 nm and the results were expressed as μg∙g^−1^ dry weight.

In the case of sucrose content, 0.2 mL of the supernatant was mixed with 0.1 mL of KOH (30%) and heated at 100 °C for 10 min. After cooling at room temperature 3 mL of anthrone (150 mg anthrone mixed with 100 mL 70% sulfuric acid) was added. Ten minutes later, the samples were cooled, and absorbance was read at 620 nm. Sucrose concentration was calculated using the standard curve and the results were expressed as μg∙g^−1^ dry weight [59].

### 2.8. Determination of Antioxidant Enzyme Activities

Leaves samples were collected and immersed immediately in liquid nitrogen followed by freeze-drying and stored at −80 °C until use. A 25 mg sample was homogenized in 5 mL 50 mM sodium phosphate buffer (pH 7.8) using a pre-chilled mortar and pestle, then centrifuged at 15,000× *g* for 20 min at 4 °C. The enzyme extract was stored at 4 °C for analysis [60]. The activity of superoxide dismutase (SOD; EC 1.15.1.1) was estimated by the method Giannopolitis and Ries (1977) [61] with slide modification. A 2 mL of reaction mixture contained 50 mM sodium phosphate buffer with 0.1 mM EDTA, 12 mM methionine, 75 µM Nitro blue tetrazolium chloride (NBT), 50 mM Na_2_CO_3_, and 100 µL enzyme extract, and in case of the blank reaction mixture, 100 µL buffer was used instead of enzyme extract. After that, 200 µL of 0.1 mM Riboflavin was added to each mixture. The tubes were shaken and irradiated under the fluorescent light (15 W) for 15 min. The absorbance of each solution was measured at 560 nm. One unit of SOD represented as the quantity of enzyme that caused 50% inhibition of NBT reduction under the experimental conditions. 

The peroxidase (POD; EC 1.11.1.7) and catalase (CAT; EC 1.11.1.6) activities were determined according to Zhang (1992) [62]. The 3 mL reaction mixture for POD consisted of 100 µL enzyme extract, 100 µL guaiacol (1.5%, *v/v*), 100 µL H_2_O_2_ (300 mM), and 2.7 mL 25 mM potassium phosphate buffer with 2 mM EDTA (pH 7.0). The increased rate of absorbance was measured spectrophotometrically at 470 nm (ε = 26.6 mM∙cm^−1^). The assay mixture for CAT contained 100 µL of enzyme extract, 100 µL of H_2_O_2_ (300 mM), and 2.8 mL of 50 mM potassium phosphate buffer with 2 mM EDTA (pH 7.0). CAT activity was assayed by observing decline absorbance reading at 240 nm (ε = 39.4 mM∙cm^−1^).

The activity of Ascorbate peroxidase (APX; EC 1.11.1.11) was assayed by the method developed by Nakano and Asada (1981) [63]. A 3 mL reaction mixture consisted of 25 µL enzyme extract, 100 µL ascorbate (7.5 mM), 100 µL H_2_O_2_ (300 mM), and 2.775 mL of 25 mM potassium phosphate buffer with 2 mM EDTA (pH 7.0). The oxidation of ascorbate was determined by the decrease of absorbance at 290 nm (ε = 2.8 mM∙cm^−1^). 

The polyphenol oxidase (PPO; EC 1.10.3.2) activity was determined following the method described by Tagele et al. (2019) [64]. The reaction mixture contained 2 mL of extract, 3 mL of 0.1M sodium phosphate buffer (pH 7.0), and 1 mL catechol (0.01M). The mixture was incubated for 5 min at 28 °C and immediately read the absorbance against a substrate blank at a wavelength of 495 nm at the one-minute interval. The activity of PPO was expressed as enzyme activity per mg dry weight per minute [65].

### 2.9. Determination of Total Phenolic Content, Total Flavonoid Content, and Antioxidant Capacity

The freeze-dried (25 mg) sample was dissolved in 10 mL of ethanol (80%, *v/v* in water) followed by sonication at 35 °C for 60 min. Afterward, the extracts were filtered (Advantech 5B filter paper, Tokoyo Roshi Kaisha Ltd., Saitama, Japan) and kept in a refrigerator (4 °C for further analysis).

Folin–Ciocalteu method was performed to estimate the total phenolic content (TPC) of the sample [66]. A reaction mixture contained 1 mL of sample, 200 µL of phenol reagent (1N), and 1.8 mL of distilled water. The mixture was vortexed and 3 min later 400 µL of Na_2_CO_3_ (10%, *v/v* in water) was added. After that, 600 µL of distilled water was added to get the final volume (4 mL) and left for 1 h incubation at room temperature. The absorbance was taken at 725 nm, and the phenolic acid was calculated from a standard calibration curve of Gallic acid and expressed as µg∙g^−1^ dry weight.

The total flavonoid content (TFC) was estimated following the method described by Adnan et al. (2019) with modifications [67]. In brief, 500 µL of the extract was mixed with 100 µL of Al(NO_3_)_3_ (10%, *w*/*v*) and 100 µL of potassium acetate (1 M) solution, and finally, 3.3 mL of distilled water was added to adjust the volume up to 4 mL. The reaction mixture was vortexed and left at room temperature for incubation for 40 min and the absorbance was measured at 415 nm by a UV-Vis spectrophotometer. The total flavonoid was measured as mg/g of Quercetin equivalent on a dry weight basis.

DPPH (2,2-diphenyl-1 picryl hydrazyl) was used to assess the antioxidant capacity of sugar beet leaf extract following the method described by Braca et al. (2003) [68]. Firstly, DPPH powder (5.914 mg) was dissolved in methanol (100 mL) to prepare a stock solution, and the absorbance range was maintained between 1.1 and 1.3 by a spectrophotometer. After that, 1 mL of extract was mixed with 3 mL of DPPH solution followed by shaking vigorously and kept in a dark room for 30 min at room temperature. The blank sample was produced by mixing distilled water (1 mL) with DPPH solution instead of extract. The absorbance was taken at 517 nm by a UV-Vis spectrophotometer (UV-180 240 V, Shimadzu Corporation, Kyoto, Japan). The scavenging capacity of the samples was calculated by using the following formula and results were expressed as percentage (%):

Inhibition (%) = [(blank sample − extract sample)/ blank sample] × 100.

### 2.10. Statistical Analysis

All results were expressed as mean ± SD (standard deviation) and mean ± SE (standard error) in tables and graphs respectively with 3 replications. All graphs were prepared by GraphPad prism 5 (San Diego, CA 92108, USA). A two-factor analysis followed by Duncan’s multiple range test (DMRT) was done by Statistix 10 (Tallahassee, FL 32312, USA). The correlation test was done by SPSS statistical software package (Ver. 23.0, SPSS Inc., Chicago, IL, USA). The least significant differences (LSD) were calculated to compare the means of different treatments with 5% level of probability.

## 3. Results and Discussions

### 3.1. Response to Drought Stress on Plant Growth Characteristics

Plant height, fresh weight, and dry weight significantly reduced (*p* < 0.05) under drought compared to the control condition in most of the genotypes (Table 2). In the case of all genotypes, plant height for control and drought conditions was found ranging from 27.67 to 18.33 cm and 21 to 16 cm, respectively. Higher plant height was recorded from S6 followed by S5, S7, S2, S3, S1, and S4 in control while S6 followed by S4, S3, S7, and S1 in drought condition. Plant fresh weight ranged from 5.94 to 3.38 g in control where the higher value was recorded for S2 followed by S9, S1, S5, and S8 genotypes. In comparison to control, drought stress condition revealed lower weight in all genotypes ranging from 2.5 to 1.11 g where S10 followed by S4, S2, S11 and S9 were recorded as a higher value. Besides, plant dry weight ranged from 0.37 to 0.17 g in control and 0.27 g to 0.13 g in drought condition. Higher plant dry weight was recorded from the genotype S2 followed by S9 and S5 in control but in case of drought, higher dry weight was recorded from the genotype S2 followed by S4, S9 and S10.

In the present experiment, cessation of watering for 10 days imposed severe water stress on all sugar beet genotypes resulted in a reduction of plant height, fresh weight and dry weight. Sugar beet is a well-known sensitive crop to water stress [6,69,70]. It is well described that drought stress can suppress seedling growth, leading to an ultimate reduction in biomass production [71,72,73]. Moreover, photosynthesis rate, partitioning coefficients and accumulations of photosynthetic products in different organs can be affected by the prolongation of drought [74]. It was also reported that drought has a negative impact on sugar beet height and dry weight [75]. Our present investigation also found a negative impact of drought stress on sugar beet growth and dry matter accumulation especially in S1, S3, S5, and S7 which might be due to the lower production of plant biomass.

### 3.2. Influence of Drought on Photosynthetic Pigments and Fluorescence

**Figure 1:**
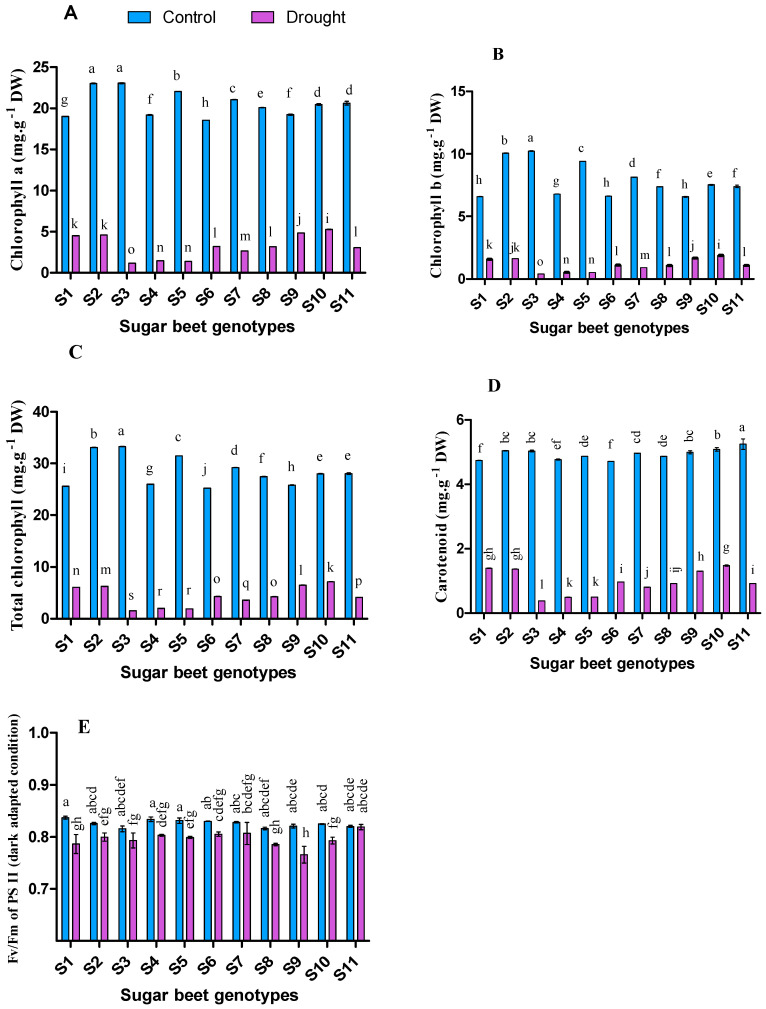
Chlorophyll a (**A**), Chlorophyll b (**B**), Total chlorophyll (**C**), Carotenoid (**D**), and Quantum yield (**E**) of 11 genotypes of sugar beet (*Beta vulgaris* L.) under control and drought conditions. Values are expressed as mean ± S.E. (*n* = 3). Different letters indicate significant differences (*p* < 0.05) among the genotypes within each parameter using Duncan’s multiple range test.

Chlorophyll a, chlorophyll b, total chlorophyll, and carotenoid significantly decreased (*p* < 0.05) in drought compared to control condition (Figure 1). In control, the genotype S3 was recorded with significantly higher chlorophyll a (23.05 mg∙g^−1^ dry weight), chlorophyll b (10.22 mg∙g^−1^ dry weight), and total chlorophyll content (33.28 mg∙g^−1^ dry weight) while the genotype S11 was detected as notable for higher Carotenoid content (5.25 mg∙g^−1^ dry weight). However, under drought condition, S10 genotype demonstrated remarkable tolerant efficiency with the highest pigments content, such as chlorophyll a (5.27 mg∙g^−1^ dry weight), chlorophyll b (1.88 mg∙g^−1^ dry weight), total chlorophyll (7.15 mg∙g^−1^ dry weight), and carotenoid (1.48 mg∙g^−1^ dry weight). Also, other significant cultivars including S2, S5, S7, S10, and S11 for control whereas S1, S2, and S9 genotypes were noteworthy for the drought stress condition. In the case of photosynthetic quantum yield analysis, no significant differences were observed among the genotypes, while it was varied significantly in drought conditions (*p* < 0.05) where S11, S7, S6, S4, S2, and S5 performed better than others.

It is well known that photosynthetic pigments considerably decrease under drought stress which causes impairment of plant growth and yield [76,77]. A low photosynthetic rate is a common issue under drought due to reduced green pigment synthesis [77]. In this study, chlorophyll a, b, total chlorophyll, and carotenoid contents significantly decreased in all the genotypes under drought conditions. However, the maximum reduction of pigments was observed in S3, S5, and S4 respectively. Importantly, activities of chlorophyllase and peroxidase are enhanced under stress conditions involved in chlorophyll breakdown that may have a crucial role in chlorophyll pigments reduction [78]. Apart from chlorophyll content and photosynthetic rate, drought has also a negative impact on different chlorophyll fluorescence parameters [79]. Concerning this principle in our study, quantum yield (Fv/Fm) was significantly reduced in most of the genotypes (except S3, S7, and S11) under 10 days’ long drought condition. It was reported that the photochemical activity of photosystem II (PS II) and electron requirement for photosynthesis both might be influenced by the drought stress, resulting in an over-excitation and photo-inhibition damage to reaction centers of PS II [80,81]. An earlier study found a significant decrease of Fv/Fm under drought conditions with comparison to the well-irrigated condition [19]. The positive correlation (Table 3) among the photosynthetic pigments, Fv/Fm and growth parameters in the present study also comply with the previous findings. 

### 3.3. Effect of Drought on Drought Tolerance Index, Relative Water Content (%), and Membrane Stability Index (%)

**Figure 2:**
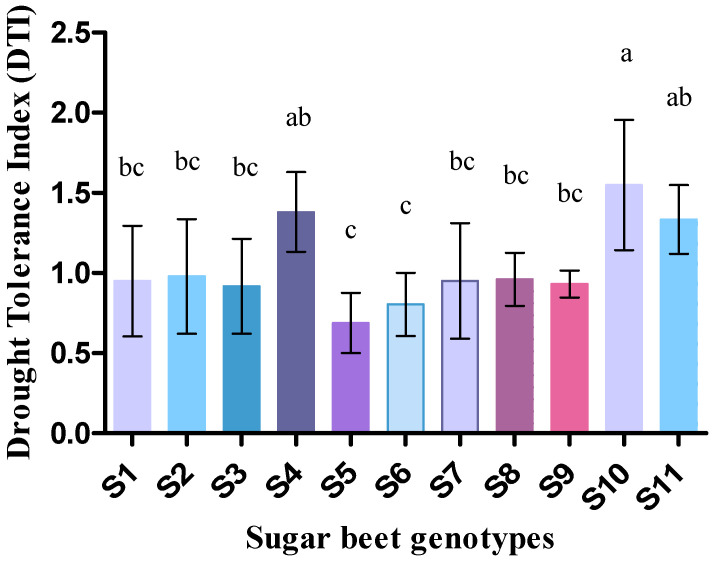
Drought tolerance index of 11 genotypes of sugar beet (*Beta vulgaris* L.) under control and drought conditions. Values are expressed as mean ± S.E. (*n* = 3). Different letters indicate significant differences (*p* < 0.05) among the genotypes within each parameter using Duncan’s multiple range test.

**Figure 3:**
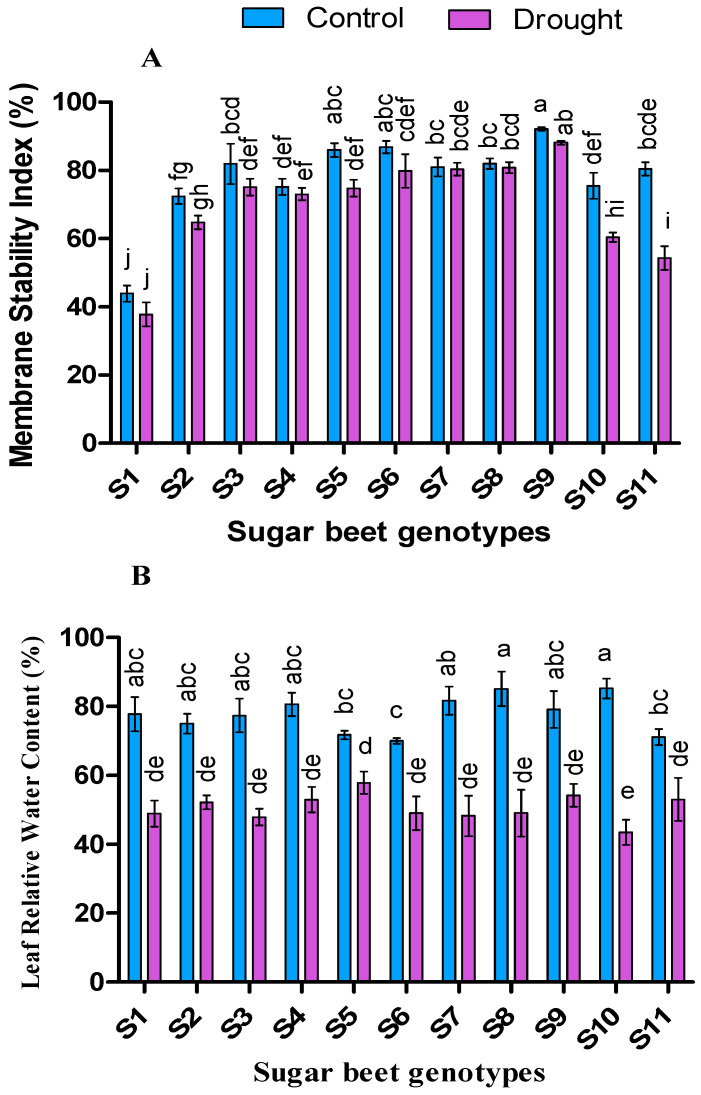
Membrane stability index (**A**) and leaf relative water content (**B**) of 11 genotypes of sugar beet (*Beta vulgaris* L.) under control and drought conditions. Values are expressed as mean ± S.E. (*n* = 3). Different letters indicate significant differences (*p* < 0.05) among the genotypes within each parameter using Duncan’s multiple range test.

Drought tolerance index (DTI) varied among the sugar beet genotypes ranging from 1.55 to 0.69 (Figure 2). The highest DTI was recorded from S10 genotype followed by S4 and S11. Relative water content (RWC %) was found higher in control (70.01 to 85.26) than drought (43.47 to 57.88) (Figure 3). Despite low statistical dissimilarities among most of the genotypes, S10 followed by S8, S7, S4, and S9 from control, and S5 followed by S9, S11, S4, and S2 from drought were found with higher RWC. Besides, membrane stability index (MSI %) was higher in control than drought condition for all the cultivars, but significant (*p* < 0.05) genotypes were noted for S5, S10, and S11. Overall, S9 genotype was recorded as higher MSI (%) in the case of both control (92.24) and drought condition (88.17) (Figure 3).

Evidence found that the cultivars with higher DTI under drought have a better carbon assimilation rate than that of lower DTI, but they may grow slowly and their productivity also hampered [52]. But in our study, the genotypes with higher DTI manifested relatively higher fresh and dry weight at the seedling stage. However, in a strict sense, this phenomenon is treated as “drought delay” not “drought tolerance” but still may be considered as a good index for early selection of suitable sugar beet genotypes under drought stress.

**Figure 4:**
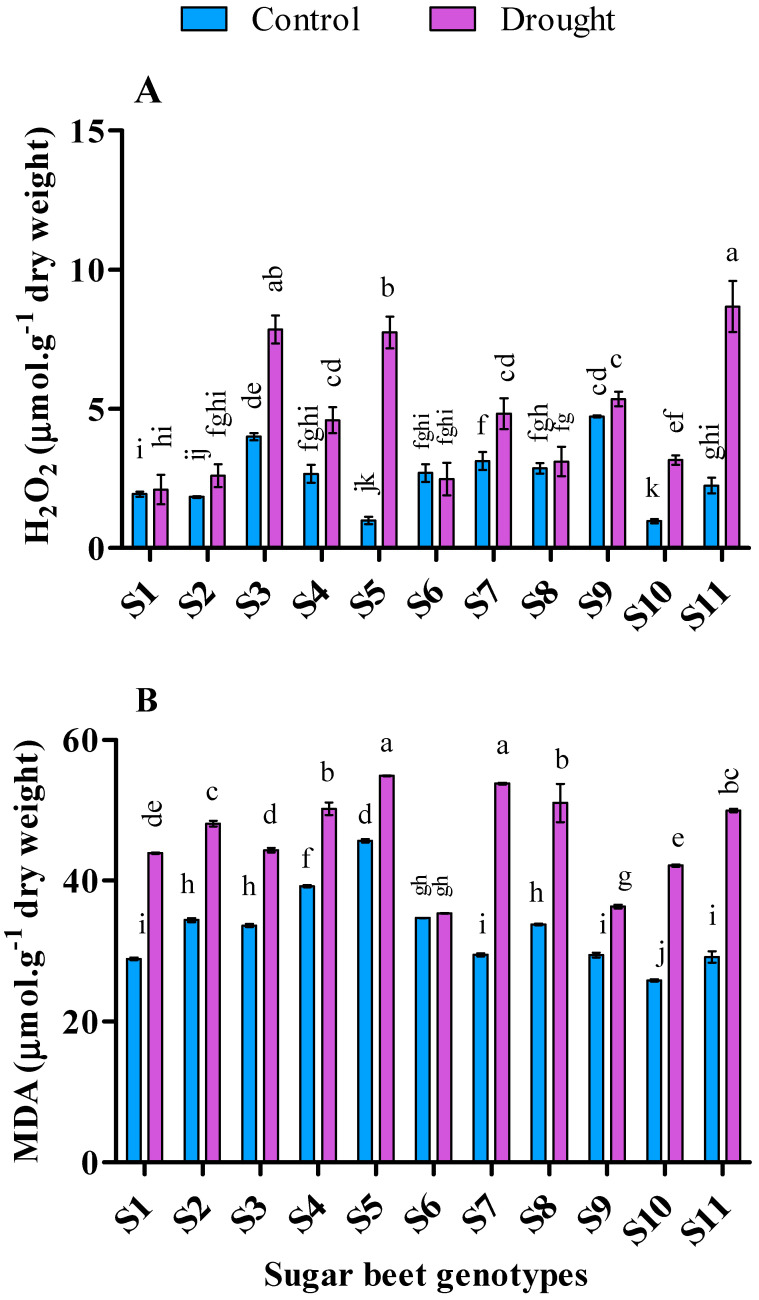
Hydrogen peroxide (**A**) and malondialdehyde content (**B**) of 11 genotypes of sugar beet (Beta vulgaris L.) under control and drought conditions. Values are expressed as mean ± S.E. (*n* = 3). Different letters indicate significant differences (*p* < 0.05) among the genotypes within each parameter using Duncan’s multiple range test.

RWC is one of the most important and reliable physiological indices by which assist to evaluate a particular genotype whether it is drought tolerant or not [69,82]. As shown in Figure 4, despite the reduction of RWC in all the genotypes under drought stress condition, the genotypes S2, S4, S5, S9, and S11 exposed as higher RWC with minimum loss (30%, 34%, 19.35%, 31.4%, and 25.4% respectively) compare to others. This higher RWC may be caused due to stomatal closure induced by stress, resulting reduction of transpiration [83,84]. To maintain cell integrity and proper functioning, the stability of cell membrane lipidome is an important index in a growing plant [85]. Like RWC, a higher membrane stability index (MSI) of tolerant genotypes may be due to reduced transpiration and efflux of solutes that are involved in the osmoregulation process of plant leaves [86]. In the present experiment the genotypes S3, S6, and S9 demonstrated strong MSI in drought conditions compared to control. Evidence showed that protecting the cell membrane helps from reducing sugar beet root yield under drought stress in different genotypes [87]. Thus, the genotypes with higher MSI under drought condition may be considered as tolerant plants.

### 3.4. Influence of Drought Stress on Lipid Peroxidation and Hydrogen Peroxide

In this experiment, almost all the experimental genotypes contained higher malondialdehyde (MDA) and hydrogen peroxide (H_2_O_2_) in drought compare to control (*p* < 0.05) (Figure 4). During drought stress, genotype S6 revealed top-most tolerance capacity confirmed by the lowest MDA (35.44 µmol∙g^−1^ dry weight) and H_2_O_2_ (2.48 µmol∙g^−1^ dry weight). Moreover, genotypes S9 (36.38 µmol∙g^−1^ dry weight), S3 (44.34 µmol∙g^−1^ dry weight), and S1 (43.91 µmol∙g^−1^ dry weight) in MDA as well as S1 (2.10 µmol∙g^−1^ dry weight), S2 (2.6 µmol∙g^−1^ dry weight), and S8 (3.1 µmol∙g^−1^ dry weight) in H_2_O_2_ demonstrated considerable drought tolerance in compare to control.

The value of the estimated MDA level is commonly used to measure the quantity of lipid peroxidation “an exponent of cell membrane damage” under drought [88]. MDA is a valuable indicator to determine the degree of damage at the cellular level caused by abiotic stress [89]. In the present study, the lower increment of MDA was recorded from S6 (1.98%) followed by S5 (20.24%), S9 (23.50%) and S3 (31.67%) and lower increment of H_2_O_2_ was recorded from S6 (−8.22%) followed by S8 (8.3%), S1 (8.7%), and S9 (13.15%). It was stated that oxidative burst induced by drought stimulate to increase MDA level, has been regarded as an index of lipid peroxidation, membrane permeability, and finally cell injury [90]. In a previous study, it was reported that, MDA folds two times higher than control in sugar beet under drought stress [91] which is in agreement with our present experiment. In contrast, over-expression of H_2_O_2_ may cause cell damage, but depend on the strong ROS scavenging mechanisms; it may act as a signaling molecule [92]. A similar experiment on *Amaranthus tricolor* [93] and fescue genotypes [29] showed that both MDA and H_2_O_2_ increased in drought conditions and they augmented progressively with the increment of drought stress. Thus, a higher generation of MDA and H_2_O_2_ can boost up the damage of cell membranes more in sensitive genotypes than tolerant genotypes. In our study, MDA and H_2_O_2_ showed a positive correlation with each other (Table 3). On the other hand, MDA manifested a positive correlation with proline, TPC and TFC, and a negative correlation with growth, photosynthetic pigments, Fv/Fm, MSI, and RWC. In this connection, higher MSI was observed in S2, S6, and S9, and higher RWC in S6 and S9 genotypes respectively with lower MDA and H_2_O_2_ accumulation.

### 3.5. Effect of Drought on Osmotic Adjustment Molecules

The sugar beet genotypes varied concerning individual osmolytes in their leaves (Table 4). Proline was found significantly higher (*p* < 0.05) in all the genotypes in drought than the control condition. A similar result was also observed for glycine betaine (GB), total soluble carbohydrate (TSC), and sucrose in most of the case. It was observed that S4, S5, S7, and S11 produced higher proline than others (*p* < 0.05), while S1, S2, S4, S6, and S11 produced higher GB and S1, S5, and S10 produced both higher TSC and sucrose under drought condition.

The accumulation of proline is a useful indicator of stress in sugar beet which can act as a signaling molecule for modulation of mitochondrial functions, influencing cell proliferation and expression of specific stress tolerant genes [94,95]. From our experiment, maximum proline increment (%) was observed from S7 (>2600%) followed by S11 (>2150%), S5 (1800%) and S3 (>1200%). Proline is considered the most important organic solute can play a key role to regulate ROS, Hydroxyl radicals, also can be an important component of cell wall proteins and that is why genotype having more proline is considered as more resistant to drought stress [58,96].

Glycine betaine also important osmoprotectants produced in response to abiotic stress which can contribute to metabolic pathways like osmotic adjustments and osmoregulation consequently reduce the negative impact [97,98]. Under osmotic stress, proline generally is accumulated in the cytosol and plays an important role in osmotic adjustment in the cytoplasm, whereas Glycine betaine is accumulated mainly in chloroplast and takes part in maintaining photosynthetic efficiency by adjustment and protection of thylakoid membrane [99,100]. Glycine betaine can also mediate osmoregulation, scavenging free radical, maintain membrane integrity, and protecting macromolecules [101]. In our study, the maximum GB increment (%) was recorded from S6 (67%) followed by S7 (51%), S11 (46%), and S9 (42%).

The production and accumulation of TSC and sucrose in the plant can be used to avoid the adverse effect of water stress [58]. In a previous study, a strong correlation was described as drought stress with the accumulation of these substances [102]. In our study, increment of TSC was found higher in S10 (68.7%), S1 (42.3%), S2 (36.9%), S11 (35.5%), and S9 (33.8%) while S10 (67.4%), S5 (66.6%), S1 (36%), S9 (22.6%), and S2 (21.8%) were found with higher increment of sucrose under drought stress.

In our study, we observed that the ROS (MDA and H_2_O_2_) were significantly increased under drought stress. Generally, the plant creates its defensive mechanism to scavenge the ROS which in this regard positively produces a higher level of osmolytes. All the osmolytes maintained a positive correlation among them (Table 3). Besides, proline mediates a positive correlation with MDA and H_2_O_2_. We also observed that GB, TSC, and sucrose have a negative correlation with photosynthetic pigments, Fv/Fm, and RWC, etc. In a previous study, it is believed that TSC and sucrose accumulation under stress depends on lower photosynthesis rate and carbohydrate consumption in plants [103]. From the overall results the genotypes S2, S6, and S11 produced higher osmolytes with higher photosynthetic traits, MSI, and RWC with minimum cell damaged by reactive oxygen species. 

### 3.6. Effect of Drought Stresses on Antioxidant Enzymes Activities

**Figure 5:**
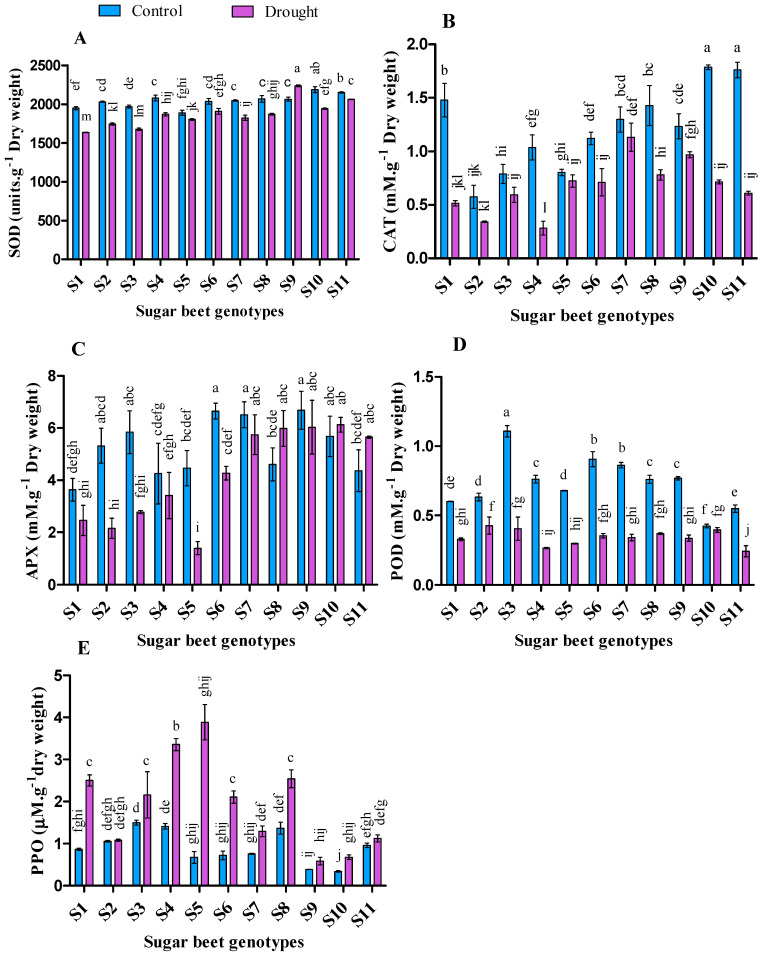
Activities of superoxide dismutase (SOD) (**A**), catalase (CAT) (**B**), ascorbate peroxidase (APX) (**C**), peroxidase (POD) (**D**), and polyphenol oxidase (PPO) (**E**) enzymes of 11 genotypes of sugar beet (*Beta vulgaris* L.) under control and drought conditions. Values are expressed as mean ± S.E. (*n* = 3). Different letters indicate significant differences (*p* < 0.05) among the genotypes within each parameter using Duncan’s multiple range test.

The activities of antioxidant enzymes varied within the variety and treatments (Figure 5). Superoxide dismutase (SOD) ranged from 2187.6 to 1888.1 (units∙g^−1^ dry weight) and 2237.5 to 1636.9 (units∙g^−1^ dry weight) in control and drought, respectively. Higher SOD was recorded from the genotypes S10 followed by S11, S4, S8, S9, and S7 in control, while genotype S9 was followed by S11, S10, S6, S8, and S4 in that of drought condition. SOD increased in S9 (8.38%) but decreased in all other genotypes after 10 days of water rescission. However, the minimum reduction of SOD under drought condition was recorded by 4.14%, 4.53%, 6.37%, and 9.57% from the genotypes S11, S5, S6, and S8, respectively.

Catalase (CAT) ranged from 1.79 to 0.57 (mM∙g^−1^ dry weight) and 1.13 to 0.28 (mM∙g^−1^ dry weight) in control and drought treatment, respectively. Higher CAT was recorded from S10 followed by S11, S1, S8, and S7 in control, while S7 was followed by S9, S8 in that of drought condition. The activity of CAT was reduced in all genotypes under drought stress compared to the control condition. However, the minimum reduction was recorded at 9.89%, 12.83%, 21.52%, 24.75%, and 36.5% from the genotypes S5, S7, S9, S3, and S6 respectively.

Ascorbate peroxidase (APX) ranged from 6.68 to 3.63 (mM∙g^−1^ dry weight) and 6.12 to 1.39 (mM∙ g^−1^ dry weight) in control and drought condition, respectively. Higher APX was recorded from S9 followed by S6, S7, S3, S10, and S2 in control, while S10 followed by S9, S8, S7, and S11 in that of drought condition. It was also observed that, only S8, S10, and S11 genotypes were found with increased APX in drought treatment compared to the control condition, while the opposite result was observed from others. Also, during peroxidase (POD) analysis, S3 (1.11 mM∙g^−1^ dry weight) followed by S6, S7, S9, S4, and S8 demonstrated higher enzyme activity in control whereas S2 (0.43 mM∙g^−1^ dry weight) followed by S3, S10, S8, and S6 in drought condition.

In the case of polyphenol oxidase (PPO), higher PPO was observed in all the genotypes in drought compared to the control condition. PPO ranged from 1.5 to 0.34 (µM∙g^−1^ dry weight) and 3.89 to 0.59 (µM∙g^−1^ dry weight) in control and drought conditions, respectively. Among all the genotypes, higher PPO was recorded for S3 followed by S4 and S8 in control, while S5 followed by S4, S8, S1, S3, and S6 in that of drought condition. Maximum augmentation in drought was observed 471.5% followed by 191.2%, 189.2%, and 138% from S5 followed by S6, S1, and S4 respectively.

Antioxidant enzyme defense activities had a high positive relation with increased abiotic stress [104]. Under drought stress, plants with higher antioxidant enzyme activities are considered to have a better free radical scavenging ability [105]. In this connection, cellular SOD acts as a first-line scavenger of ROS by catalyzing the superoxide radical (O_2_^−^^•^) into oxygen and H_2_O_2_ [106,107]. It was stated that, prolong drought stress causes oxidative damage as exhibited by a general decline in antioxidant enzyme activities and an increase in lipid peroxidation [108]. In our study, higher lipid peroxidation was found with lower antioxidant activities in most cases. Generally, higher SOD activity may be exerted due to the consequences of excess superoxide radical generation [29] that can act as a signal for the induction of antioxidant enzyme, resulting in greater SOD induction [109]. This hypothesis indicated that the genotypes S9 followed by S11, S10, S6 and S8 with comparatively higher SOD might have a high capacity to catalyze superoxide radical under drought conditions.

Besides, CAT, APX, and POD are also the major enzymes involved in the detoxification of H_2_O_2_ (produced through the dismutation of O_2_^−^^•^ in peroxisomes and chloroplasts) to H_2_O and O_2_ [29,110,111]. In the present experiment, genotypes S6, S7, S8, S9, and S10 were found with higher CAT, APX, and POD activity under drought stress. This may be attributed to the increased H_2_O_2_ and MDA accumulation, resulting in excitation of enzyme activities. A similar response was also observed in previous research on cotton [112]. Usually, high oxidative stress provokes the H_2_O_2_ generation under drought stress which also stimulates to produce high antioxidant enzyme activity. Subsequently, this enzyme activity may minimize the adverse effect of abiotic stresses either by acting as a bio-signal and/or modulating resistant gene expression [113]. Moreover, It has been reported that PPO can protect the over reduction of photosynthetic electron transport during environmental stress [114]. In a previous study, PPO activity was found significantly high in white clover after 7 days of drought treatment [115]. However, some conflict results were also reported, which showed that suppression of PPO activity increased drought tolerance in tomato [116]. In our study, we observed a positive correlation of PPO with TPC (Table 3). In this connection, the genotypes S1, S3, S5, S6, and S8 performed better in drought conditions. Besides, PPO can directly influence photosynthesis by acting as an oxygen buffer to facilitate reactive oxygen scavenging [114,117,118]. Although PPO regulates available oxygen to oxidize phenolic compounds to o-quinones and H_2_O, but it can regenerate o-diphenol to o-quinones by providing a source of reducing power with the close association of photosystems during photosynthesis [118,119,120].

In general, under stress conditions plant physiological activities and performance decline with a decrease in antioxidant enzymes activities and an increase in ROS chemicals. These phenomena indicating an inability of plant cells against ROS chemicals. The activities and involvement of different antioxidant enzymes in ROS scavenging vary with some factors including plant species, stress severity and duration [108]. Sairam et al. (2000) observed a variation of antioxidant enzymes activities such as SOD, CAT, and APX among different wheat genotypes [121]. In the present study, we observed lower activities of SOD in S5, CAT in S2, S4, S5, S6, and S8, APX in S4 and S5, POD in S4, S5 and S11, and PPO in S7 and S11 genotypes along with a higher accumulation of MDA. From the overall observations the genotypes S6, S7, S8, S9, and S10 showed better stress response through redox homeostasis process by using different antioxidant enzymes under drought condition. 

### 3.7. Influence of Drought Stresses on Antioxidant Activities

**Figure 6:**
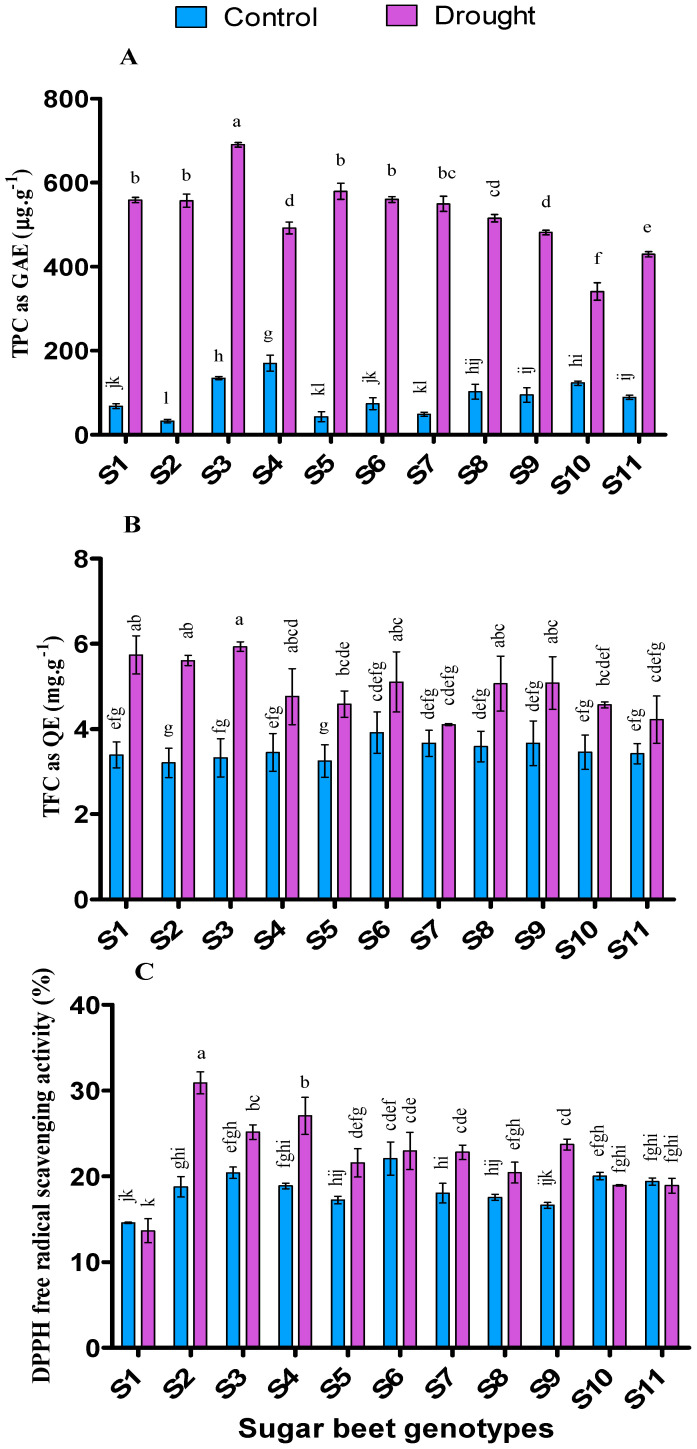
Total polyphenol content (TPC) (**A**), total flavonoid content (TFC) (**B**) and DPPH radical scavenging activity (%) (**C**) of 11 genotypes of sugar beet (*Beta vulgaris* L.) under control and drought conditions. Values are expressed as mean ± S.E. (*n* = 3). Different letters indicate significant differences (*p* < 0.05) among the genotypes within each parameter using Duncan’s multiple range test.

Total phenolic content (TPC) was augmented in all the genotypes under drought stress (*p* < 0.05) in the present experiment (Figure 6). TPC ranged from 170.37 to 32.59 (µg∙g^−1^ as GAE) and 690.37 to 340.74 (µg∙g^−1^ as GAE) in control and drought, respectively. Higher TPC was recorded from S4 followed by S3, S10, S8, S9, and S11 in control, while S3 was followed by S5, S6, S1, S2, and S7 in that of drought condition.

Total flavonoid content (TFC) is also influenced by drought stress in all the genotypes compared to control (*p* < 0.05) (Figure 6). TFC ranged from 3.92 to 3.21 (mg∙g^−1^ as QE) and 5.93 to 4.11 (mg∙g^−1^ as QE) in control and drought treatment. Higher TFC was recorded from S6 followed by S9, S7, and S8 in control, while S3 was followed by S1, S2, S6, S9, S8, and S4 in that of drought condition.

Antioxidant capacity (DPPH) was observed higher under drought treatment than the control condition (*p* < 0.05) in most of the genotypes (except S1, S10, and S11) (Figure 6). DPPH radical scavenging activity (%) ranged from 22% to 14.58% and 30.91% to 13.65% in control and drought, respectively. Higher activity was observed from S6 followed by S3, S10, S11, and S4 in control while S2 followed by S4, S3, S9, S6, S7, and S5 in drought condition. 

Both TPC and TFC were found higher under drought stress compared to control in all the cultivars. In a previous experiment, it was observed that TPC, TFC, and total antioxidant activity were significantly increased with the severity of drought stress in Amaranthus leafy vegetables [122]. In our experiment, higher augmentation of TPC were observed in S2 (1600%), S5 (1250%), S7 (1025%), and S1 (720%); higher TFC augmented in S3 (78%), S2 (75%), and S1 (69%) while higher DPPH activity were found in S2 (64%) followed by S4 (43%), S9 (73%), S7 (26%), and S5 (25%) under drought stress. In general, the accumulation of ROS under biotic and abiotic stress stimulates to enhance the antioxidant properties of plants [123]. Polyphenols can scavenge reactive oxygen species and may modulate MDA by controlling lipid alkoxyl radical. On the other hand, peroxidase can oxidize flavonoids and phenylpropanoids, resulting in H_2_O_2_-scavenging in the phenolic/ASA/POD system [93].

Generally, total antioxidant activity is the combined activities of both enzymatic and non-enzymatic antioxidants in plants. In a previous experiment, total antioxidant activity along with total polyphenol and total flavonoid content significantly increased in *Achillea* species [124] and also in soybean [125] by drought stress. Tolerant plants can protect themselves by augmenting the antioxidant enzymes, molecules activity, and quantity under stress (biotic or abiotic) condition [93]. A plant having higher antioxidants can improve their defensive responses under oxidative stress by scavenging free radicals rapidly [126]. Since drought can trigger the increment of ROS, hence a higher amount of antioxidants are required to get acclimated by improving the tolerance level [127]. Antioxidant activity plays a vital role in plant defense mechanisms by keeping the balance between synthesis and scavenging of free radicals [128]. In our study, antioxidants have a positive correlation with osmolytes however, a vice-versa relationship was also observed with enzymes (Table 3). Overall, the genotypes S2, S3, S5, S6, S7, and S9 performed better regarding TPC, TFC, and antioxidant capacity.

## 4. Conclusions

Under drought conditions, seedlings growth and development, photosynthetic pigments and photosynthetic quantum yield (Fv/Fm) expressively reduced compare to control in all sugar beet genotypes. Significant reductions were also observed in membrane stability index, leaf relative water content, and activity of antioxidant enzymes. Positive correlations were noticed between growth parameters and photosynthetic traits, physiological parameters and antioxidant enzyme activities respectively. On the other hand, drought significantly increased the reactive oxygen species and osmotic adjusting molecules in sugar beet seedlings. A remarkable increment of secondary metabolites and antioxidant capacity was also observed in drought conditions. Although enzymes have a negative correlation with osmolytes and secondary metabolites, the reactive oxygen species maintained a positive correlation with them. Based on the results, higher activities of antioxidant enzymes, osmolytes, membrane stability index and relative water content with lower reactive oxygen species accumulation were the most important factors considering drought-tolerant sugar beet seedlings. After analyzing all the data, the cultivars S2 (HELENIKA), S6 (RECODDINA), S7 (GREGOIA), S8 (SV2347), S9 (SV2348), S10 (BSRI Sugarbeet 1), and S11 (BSRI Sugarbeet 2) were found best fitted under drought condition. The findings of the experiment can help further for better understanding of different mechanisms related to the adaptability of sugar beet in drought conditions to improve drought tolerance cultivars.

## Figures and Tables

**Table 1:** Sugar beet cultivars used in the experiment.

S.N.	Name of the Cultivar	Origin	Code Name
**1**	MAXIMELLA	Germany	S1
**2**	HELENIKA	Germany	S2
**3**	TERRANOVA	Germany	S3
**4**	TOLERANZA	Germany	S4
**5**	BORNITA	Germany	S5
**6**	RECODDINA	Germany	S6
**7**	GREGOIA	Germany	S7
**8**	SV2347	Belgium	S8
**9**	SV2348	Belgium	S9
**10**	BSRI Sugarbeet 1	Bangladesh	S10
**11**	BSRI Sugarbeet 2	Bangladesh	S11

S.N.: Serial number.

**Table 2:** Plant height, fresh weight and dry weight of 11 genotypes of sugar beet (*Beta vulgaris* L.) under control and drought conditions.

Cultivars	Plant Height (cm)		Fresh Weight/Plant (gm)		Dry Weight/Plant (gm)	
	Control	Drought	Control	Drought	Control	Drought
**S1**	23.33 ± 1.53 bc	17.67 ± 0.58 de	5.09 ± 1.52 abc	1.72 ± 0.38 gh	0.28 ± 0.09 bcd	0.19 ± 0.03 eh
**S2**	24.33 ± 1.53 bc	17.17 ± 0.29 e	5.94 ± 0.77 a	1.99 ± 0.43 gh	0.36 ± 0.04 a	0.27 ± 0.09 b–e
**S3**	23.83 ± 3.82 bc	18.33 ± 1.15 de	3.38 ± 1.20 ef	1.10 ± 0.13 h	0.20 ± 0.09 d–h	0.13 ± 0.04 h
**S4**	23.33 ± 3.79 bc	18.43 ± 3.19 de	4.00 ± 0.50 cde	2.07 ± 0.41 gh	0.25 ± 0.02 b–f	0.23 ± 0.02 c–g
**S5**	25.00 ± 1.00 ab	17.17 ± 2.93 e	4.72 ± 1.30 bcd	1.38 ± 0.42 gh	0.28 ± 0.06 abc	0.14 ± 0.03 h
**S6**	27.67 ± 0.76 a	21.00 ± 3.04 cd	4.55 ± 1.51 cde	1.38 ± 0.30 gh	0.26 ± 0.08 b–e	0.15 ± 0.02 gh
**S7**	24.67 ± 2.08 ab	18.17 ± 2.75 de	4.12 ± 1.12 cde	1.71 ± 0.29 gh	0.26 ± 0.08 b–e	0.18 ± 0.05 fgh
**S8**	18.67 ± 0.58 de	17.00 ± 2.18 e	4.71 ± 0.19 bcd	1.79 ± 0.37 gh	0.27 ± 0.005 b–e	0.19 ± 0.02 e–h
**S9**	18.33 ± 1.53 de	17.33 ± 2.25 e	5.88 ± 0.12 ab	1.85 ± 0.06 gh	0.33 ± 0.03 ab	0.23 ± 0.01 c–g
**S10**	18.10 ± 0.36 de	16.67 ± 1.53 e	4.18 ± 0.19 cde	2.50 ± 0.29 fg	0.23 ± 0.01 c–g	0.23 ± 0.01 c–g
**S11**	19.33 ± 2.02 de	16.00 ± 1.73 e	3.54 ± 0.53 def	1.86 ± 0.37 gh	0.18 ± 0.02 gh	0.18 ± 0.03 fgh
**LSD _(0.05)_**	3.49		1.18		0.08	

Different letters indicate significant differences (*p* < 0.05) among the genotypes within each parameter. Values are expressed as mean ± SD (*n* = 3).

**Table 3:** Correlations among the parameters in 11 genotypes of sugar beet (*Beta vulgaris* L.) under control and drought conditions.

	DTI	Chl a	Chl b	Car	RWC	MSI	MDA	Fv/Fm	Plant ht.	Plant fr. Wt.	Plant dry wt.	H2O2	SOD	CAT	APX	POD	PPO	Proline	GB	TSC	Sucrose	TPC	TFC	DPPH
**DTI**	1																							
**Chl a**	0.005	1																						
**Chl b**	−0.036	0.988 **	1																					
**Car**	0.038	0.994 **	0.967 **	1																				
**RWC**	0.025	0.926 **	0.893 **	0.932 **	1																			
**MSI**	−0.256	0.276	0.282	0.262	0.286	1																		
**MDA**	−0.133	−0.758 **	−0.705 **	−0.786 **	−0.728 **	−0.176	1																	
**Fv/Fm**	0.073	0.767 **	0.739 **	0.772 **	0.762 **	0.065	−0.463 *	1																
**Plant ht.**	−0.333	0.699 **	0.721 **	0.669 **	0.570 **	0.239	−0.444 *	0.681 **	1															
**Plant fr. Wt.**	−0.142	0.709 **	0.731 **	0.676 **	0.606 **	0.125	−0.374	0.717 **	0.927 **	1														
**Plant dry wt.**	−0.202	0.391	0.42	0.356	0.315	−0.026	−0.105	0.495 *	0.811 **	0.90 **	1													
**H2O2**	−0.079	−0.580 **	−0.565 **	−0.587 **	−0.426 *	0.015	0.498 *	−0.365	−0.465 *	−0.429 *	−0.346	1												
**SOD**	0.365	0.560 **	0.514 *	0.582 **	0.595 **	0.415	−0.643 **	0.342	0.197	0.265	−0.05	−0.174	1											
**CAT**	0.178	0.609 **	0.518 *	0.659 **	0.668 **	0.257	−0.693 **	0.465 *	0.194	0.151	−0.134	−0.343	0.640 **	1										
**APX**	0.18	0.391	0.38	0.392	0.285	0.450 *	−0.414	0.204	0.253	0.219	−0.024	−0.141	0.648 **	0.385	1									
**POD**	−0.229	0.831 **	0.835 **	0.817 **	0.752 **	0.388	−0.584 **	0.604 **	0.760 **	0.713 **	0.492 *	−0.356	0.332	0.36	0.406	1								
**PPO**	−0.197	−0.596 **	−0.558 **	−0.614 **	−0.461 *	−0.175	0.625	−0.412	−0.301	−0.329	−0.045	0.389	−0.610 **	−0.531 *	−0.642 **	−0.429 *	1							
**Proline**	−0.054	−0.867 **	−0.840 **	−0.873 **	−0.744 **	−0.282	0.835 **	−0.530*	−0.594 **	−0.590 **	−0.293	0.606 **	−0.589 **	−0.561 **	−0.484 *	−0.750 **	0.704 **	1						
**GB**	0.094	−0.366	−0.301	−0.404	−0.443 *	−0.513 *	0.42	−0.231	−0.177	−0.071	0.108	−0.059	−0.461 *	−0.702 **	−0.539 **	−0.473 *	0.347	0.295	1					
**TSC**	−0.327	−0.500 *	−0.480 *	−0.507 *	−0.494 *	−0.440 *	0.355	−0.583 **	−0.266	−0.275	−0.023	0.205	−0.590 **	−0.470 *	−0.428 *	−0.243	0.364	0.33	0.221	1				
**Sucrose**	−0.402	−0.425 *	−0.416	−0.419	−0.486 *	−0.268	0.278	−0.535 *	−0.203	−0.276	−0.114	0.188	−0.503 *	−0.276	−0.288	−0.185	0.226	0.296	−0.011	0.801 **	1			
**TPC**	−0.093	−0.964 **	−0.941 **	−0.965 **	−0.885 **	−0.235	0.729 **	−0.790 **	−0.671 **	−0.692 **	−0.354	0.553 **	−0.634 **	−0.616 **	−0.486 *	−0.769 **	0.634 **	0.842 **	0.375	0.533 *	0.462 *	1		
**TFC**	−0.132	−0.863 **	−0.855 **	−0.853 **	−0.832 **	−0.275	0.534 *	−0.820 **	−0.757 **	−0.636 **	−0.301	0.377	−0.635 **	−0.617 **	−0.460 *	−0.645 **	0.501 *	0.618 **	0.391	0.660 **	0.536 *	0.903 **	1	
**DPPH**	0.003	−0.506 *	−0.478 *	−0.517 *	−0.455 *	0.274	0.403	−0.391	−0.245	−0.275	−0.088	0.291	−0.221	−0.436 *	−0.25	−0.314	0.253	0.423 *	0.217	−0.031	0.035	0.549 **	0.506 *	1

* Indicates statistical difference significance at *p* < 0.05 among the treatments by Duncan’s multiple range tests; ** Indicates statistical difference significance at *p* < 0.01 among the treatments by Duncan’s multiple range tests.

**Table 4:** Osmolytes of 11 genotypes of sugar beet (*Beta vulgaris* L.) under control and drought conditions.

Genotypes	Proline (μg∙g^−1^ DW)		Glycine Betaine (mg∙g^−1^ DW)		TSC (μg∙g^−1^ DW)		Sucrose (μg∙g^−1^ DW)	
	Control	Drought	Control	Drought	Control	Drought	Control	Drought
**S1**	12.99 ± 0.44 h	159.45 ± 0.16 c	7.35 ± 0.05 g	8.37 ± 0.03 d	471.33 ± 13.87 ghi	670.67 ± 14.39 a	151.17 ± 1.39 e	205.59 ± 4.48 a
**S2**	9.70 ± 0.44 h	119.01 ± 12.87 d	8.91 ± 0.06 b	8.78 ± 0.06 c	389.44 ± 63.57 jkl	533.00 ± 2.17 de	142.94 ± 0.30 f	174.09 ± 3.12 c
**S3**	8.89 ± 0.22 h	118.36 ± 15.39 d	5.91 ± 0.06 k	7.66 ± 0.06 f	456.22 ± 24.94 hij	547.22 ± 18.17 cd	149.72 ± 7.19 e	172.77 ± 4.07 cd
**S4**	18.92 ± 2.35 h	217.99 ± 15.21 b	6.78 ± 0.08 i	9.10 ± 0.1 a	444.11 ± 40.21 hij	388.44 ± 38.83 l	127.51 ± 1.29 g	96.15 ± 2.79 j
**S5**	12.41 ± 2.69 h	235.43 ± 0.21 a	8.34 ± 0.07 d	6.14 ± 0.05 j	445.00 ± 9.75 h–k	579.67 ± 11.07 bc	124.25 ± 1.59 gh	206.98 ± 5.08 a
**S6**	8.20 ± 1.22 h	102.49 ± 2.30 e	4.61 ± 0.05 o	7.78 ± 0.07 e	435.22 ± 17.25 h–k	496.78 ± 2.17 fg	173.37 ± 2.58 c	154.44 ± 0.40 e
**S7**	8.56 ± 0.71 h	233.46 ± 3.30 a	3.09 ± 0.04 q	4.72 ± 0.05 n	496.78 ± 26.34 efg	416.11 ± 2.80 kl	139.02 ± 2.99 f	167.28 ± 0.41 d
**S8**	16.25 ± 0.34 h	90.73 ± 7.13 e	5.36 ± 0.05 l	7.12 ± 0.05 h	501.67 ± 18.15 ef	477.33 ± 8.27 fgh	139.35 ± 0.60 f	149.74 ± 2.78 e
**S9**	12.70 ± 4.62 h	75.75 ± 12.19 f	4.31 ± 0.06 p	6.16 ± 0.04 j	394.56 ± 10.56 l	527.89 ± 27.71 de	140.28 ± 8.35 f	172.04 ± 3.87 cd
**S10**	10.52 ± 0.09 h	59.07 ± 4.83 g	5.84 ± 0.06 k	7.29 ± 0.04 g	347.22 ± 27.74 m	585.78 ± 3.89 b	117.06 ± 2.70 i	195.93 ± 2.78 b
**S11**	7.62 ± 0.61 h	172.39 ± 22.07 c	5.24 ± 0.05 m	7.76 ± 0.06 e	320.11 ± 10.24 m	433.89 ± 25.32 ijk	125.26 ± 8.38 g	119.04 ± 1.79 hi
**LSD _(0.05)_**	13.43		0.097		34.16		6.08	

Different letters indicate significant differences (*p* < 0.05) among the genotypes within each parameter. Values are expressed as mean ± SD (*n* = 3).

## References

[1] He M., He C.-Q., Ding N.-Z. (2018). Abiotic stresses: General defenses of land plants and chances for engineering multistress tolerance. Front. Plant Sci..

[2] Jeandroz S., Lamotte O. (2017). Plant responses to biotic and abiotic stresses: Lessons from cell signaling. Front. Plant Sci..

[3] Qin F., Shinozaki K., Yamaguchi-Shinozaki K. (2011). Achievements and challenges in understanding plant abiotic stress responses and tolerance. Plant Cell Physiol..

[4] Luković J., Maksimović I., Zorić L., Nagl N., Perčić M., Polić D., Putnik-Delić M. (2009). Histological characteristics of sugar beet leaves potentially linked to drought tolerance. Ind. Crops Prod..

[5] Islam J., Choi S.P., Azad O.K., Kim J.W., Lim Y.-S. (2020). Evaluation of tuber yield and marketable quality of newly developed thirty-two potato varieties grown in three different ecological zones in South Korea. Agriculture.

[6] Chołuj D., Wiśniewska A., Szafrański K.M., Cebula J., Gozdowski D., Podlaski S. (2014). Assessment of the physiological responses to drought in different sugar beet genotypes in connection with their genetic distance. J. Plant Physiol..

[7] Sadeghian S.Y., Fazli H., Mohammadian R., Taleghani D.F., Mesbah M. (2000). Genetic variation for drought stress in sugarbeet. J. Sugar Beet Res..

[8] Zhang L., Zhou T. (2015). Drought over East Asia: A review. J. Clim..

[9] Kwon H., Lall U., Kim S. (2016). The unusual 2013–2015 drought in South Korea in the context of a multicentury precipitation record: Inferences from a nonstationary, multivariate, Bayesian copula model. Geophys. Res. Lett..

[10] Kim C.J., Park M.J., Lee J.H. (2014). Analysis of climate change impacts on the spatial and frequency patterns of drought using a potential drought hazard mapping approach. Int. J. Climatol..

[11] Schuppler U., He P.-H., John P.C.L., Munns R. (1998). Effect of water stress on cell division and Cdc2-like cell cycle kinase activity in wheat leaves. Plant Physiol..

[12] Jaleel C.A., Manivannan P., Lakshmanan G.M.A., Gomathinayagam M., Panneerselvam R. (2008). Alterations in morphological parameters and photosynthetic pigment responses of *Catharanthus roseus* under soil water deficits. Colloids Surf. B Biointerfaces.

[13] Jaleel C.A., Gopi R., Manivannan P., Gomathinayagam M., Sridharan R., Panneerselvam R. (2008). Antioxidant potential and indole alkaloid profile variations with water deficits along different parts of two varieties of *Catharanthus roseus*. Colloids Surf. B Biointerfaces.

[14] Rampino P., Pataleo S., Gerardi C., Mita G., Perrotta C. (2006). Drought stress response in wheat: Physiological and molecular analysis of resistant and sensitive genotypes. Plant. Cell Environ..

[15] Nouri A., Etminan A., Teixeira da Silva J.A., Mohammadi R. (2011). Assessment of yield, yield-related traits and drought tolerance of durum wheat genotypes (*Triticum turgidum* var. *durum* Desf.). Aust. J. Crop Sci..

[16] Fukai S., Fischer K.S. (2012). Field phenotyping strategies and breeding for adaptation of rice to drought. Front. Physiol..

[17] Munjal R., Dhanda S.S. (2016). Assessment of drought resistance in Indian wheat cultivars for morpho-physiological traits. Ekin J. Crop Breed. Genet..

[18] Lebaudy A., Vavasseur A., Hosy E., Dreyer I., Leonhardt N., Thibaud J.-B., Véry A.-A., Simonneau T., Sentenac H. (2008). Plant adaptation to fluctuating environment and biomass production are strongly dependent on guard cell potassium channels. Proc. Natl. Acad. Sci. USA.

[19] Nemeskéri E., Neményi A., Bőcs A., Pék Z., Helyes L. (2019). Physiological factors and their relationship with the productivity of processing tomato under different water supplies. Water.

[20] Nemeskéri E., Molnár K., Pék Z., Helyes L. (2018). Effect of water supply on the water use-related physiological traits and yield of snap beans in dry seasons. Irrig. Sci..

[21] Mundim F.M., Pringle E.G. (2018). Whole-plant metabolic allocation under water stress. Front. Plant Sci..

[22] Chaves M.M., Oliveira M.M. (2004). Mechanisms underlying plant resilience to water deficits: Prospects for water-saving agriculture. J. Exp. Bot..

[23] Chan K.X., Wirtz M., Phua S.Y., Estavillo G.M., Pogson B.J. (2013). Balancing metabolites in drought: The sulfur assimilation conundrum. Trends Plant Sci..

[24] Zivcak M., Brestic M., Sytar O. (2016). Osmotic adjustment and plant adaptation to drought stress. Drought Stress Tolerance in Plants.

[25] Nemeskeri E., Kovacs-Nagy E., Nyeki J., Sardi E. (2015). Responses of apple tree cultivars to drought: Carbohydrate composition in the leaves. Turk. J. Agric. For..

[26] Guo R., Shi L., Jiao Y., Li M., Zhong X., Gu F., Liu Q., Xia X., Li H. (2018). Metabolic responses to drought stress in the tissues of drought-tolerant and drought-sensitive wheat genotype seedlings. AoB Plants.

[27] Zabalza A., Gálvez L., Marino D., Royuela M., Arrese-Igor C., González E.M. (2008). The application of ascorbate or its immediate precursor, galactono-1, 4-lactone, does not affect the response of nitrogen-fixing pea nodules to water stress. J. Plant Physiol..

[28] Smirnoff N. (1993). Tansley review no. 52. The role of active oxygen in the response of plants to water deficit and desiccation. New Phytol..

[29] Bi A., Fan J., Hu Z., Wang G., Amombo E., Fu J., Hu T. (2016). Differential acclimation of enzymatic antioxidant metabolism and photosystem II photochemistry in tall fescue under drought and heat and the combined stresses. Front. Plant Sci..

[30] Signorelli S., Corpas F.J., Borsani O., Barroso J.B., Monza J. (2013). Water stress induces a differential and spatially distributed nitro-oxidative stress response in roots and leaves of *Lotus japonicus*. Plant Sci..

[31] Noctor G., Mhamdi A., Foyer C.H. (2014). The roles of reactive oxygen metabolism in drought: Not so cut and dried. Plant Physiol..

[32] Scandalios J.G., Guan L., Polidoros A.N. (1997). Catalases in plants: Gene structure, properties, regulation, and expression. Cold Spring Harb. Monogr. Ser..

[33] Ali B., Hasan S.A., Hayat S., Hayat Q., Yadav S., Fariduddin Q., Ahmad A. (2008). A role for brassinosteroids in the amelioration of aluminium stress through antioxidant system in mung bean (*Vigna radiata* L. Wilczek). Environ. Exp. Bot..

[34] Rubio M.C., González E.M., Minchin F.R., Webb K.J., Arrese-Igor C., Ramos J., Becana M. (2002). Effects of water stress on antioxidant enzymes of leaves and nodules of transgenic alfalfa overexpressing superoxide dismutases. Physiol. Plant..

[35] Jiang M., Zhang J. (2002). Water stress-induced abscisic acid accumulation triggers the increased generation of reactive oxygen species and up-regulates the activities of antioxidant enzymes in maize leaves. J. Exp. Bot..

[36] Chen F., Liu C.-J., Tschaplinski T.J., Zhao N. (2009). Genomics of secondary metabolism in *Populus*: Interactions with biotic and abiotic environments. Crit. Rev. Plant Sci..

[37] Ncube B., Van Staden J. (2015). Tilting plant metabolism for improved metabolite biosynthesis and enhanced human benefit. Molecules.

[38] Zhao J., Davis L.C., Verpoorte R. (2005). Elicitor signal transduction leading to production of plant secondary metabolites. Biotechnol. Adv..

[39] Islam M.J., Hassan M.K., Sarker S.R., Rahman A.B., Fakir M.S.A. (2017). Light and temperature effects on sprout yield and its proximate composition and vitamin C content in *lignosus* and mung beans. J. Bangladesh Agric. Univ..

[40] Bharti N., Yadav D., Barnawal D., Maji D., Kalra A. (2013). *Exiguobacterium oxidotolerans*, a halotolerant plant growth promoting rhizobacteria, improves yield and content of secondary metabolites in *Bacopa monnieri* (L.) Pennell under primary and secondary salt stress. World J. Microbiol. Biotechnol..

[41] Pourcel L., Routaboul J.-M., Cheynier V., Lepiniec L., Debeaujon I. (2007). Flavonoid oxidation in plants: From biochemical properties to physiological functions. Trends Plant Sci..

[42] Ferrat L., Pergent-Martini C., Roméo M. (2003). Assessment of the use of biomarkers in aquatic plants for the evaluation of environmental quality: Application to seagrasses. Aquat. Toxicol..

[43] Abo-Elwafa S.F., Abdel-Rahim H.M., Abou-Salama A.M., Teama E.A. (2006). Sugar beet floral induction and fertility: Effect of vernalization and day-length extension. Sugar Tech.

[44] Brar N.S., Dhillon B.S., Saini K.S., Sharma P.K. (2015). Agronomy of sugar beet cultivation—A review. Agric. Rev..

[45] Mutasa-Göttgens E.S., Qi A., Zhang W., Schulze-Buxloh G., Jennings A., Hohmann U., Müller A.E., Hedden P. (2010). Bolting and flowering control in sugar beet: Relationships and effects of gibberellin, the bolting gene B and vernalization. AoB Plants.

[46] Publication. https://web.kma.go.kr/eng/aboutkma/webzine.jsp.

[47] Sunrise and sunset in South Korea. https://www.worlddata.info/asia/south-korea/sunset.php.

[48] Lichtenthaler H.K. (1987). Chlorophylls and carotenoids: Pigments of photosynthetic biomembranes. Methods in Enzymology.

[49] Meng L.-L., Song J.-F., Wen J., Zhang J., Wei J.-H. (2016). Effects of drought stress on fluorescence characteristics of photosystem II in leaves of *Plectranthus scutellarioides*. Photosynthetica.

[50] Barrs H.D., Weatherley P.E. (1962). A re-examination of the relative turgidity technique for estimating water deficits in leaves. Aust. J. Biol. Sci..

[51] Fernandez G.C.J. Effective selection criteria for assessing plant stress tolerance. Proceedings of the International Symposium on Adaptation of Vegetables and other Food Crops in Temperature and Water Stress.

[52] Ober E.S., Clark C.J.A., Le Bloa M., Royal A., Jaggard K.W., Pidgeon J.D. (2004). Assessing the genetic resources to improve drought tolerance in sugar beet: Agronomic traits of diverse genotypes under droughted and irrigated conditions. F. Crop. Res..

[53] Sairam R.K., Rao K.V., Srivastava G.C. (2002). Differential response of wheat genotypes to long term salinity stress in relation to oxidative stress, antioxidant activity and osmolyte concentration. Plant Sci..

[54] Heath R.L., Packer L. (1968). Photoperoxidation in isolated chloroplasts: I. Kinetics and stoichiometry of fatty acid peroxidation. Arch. Biochem. Biophys..

[55] Singh N., Ma L.Q., Srivastava M., Rathinasabapathi B. (2006). Metabolic adaptations to arsenic-induced oxidative stress in *Pteris vittata* L. and *Pteris ensiformis* L.. Plant Sci..

[56] Bates L.S., Waldren R.P., Teare I.D. (1973). Rapid determination of free proline for water-stress studies. Plant Soil.

[57] Xu Z., Sun M., Jiang X., Sun H., Dang X., Cong H., Qiao F. (2018). Glycinebetaine biosynthesis in response to osmotic stress depends on jasmonate signaling in watermelon suspension cells. Front. Plant Sci..

[58] Khoyerdi F.F., Shamshiri M.H., Estaji A. (2016). Changes in some physiological and osmotic parameters of several pistachio genotypes under drought stress. Sci. Hortic. (Amsterdam).

[59] Van Handel E. (1968). Direct microdetermination of sucrose. Anal. Biochem..

[60] Slabbert M.M., Krüger G.H.J. (2014). Antioxidant enzyme activity, proline accumulation, leaf area and cell membrane stability in water stressed *Amaranthus* leaves. South Afr. J. Bot..

[61] Giannopolitis C.N., Ries S.K. (1977). Superoxide dismutases: I. Occurrence in higher plants. Plant Physiol..

[62] Zhang X.Z. (1992). The measurement and mechanism of lipid peroxidation and SOD, POD and CAT activities in biological system. Res. Methodol. Crop Physiol. Agric. Press. Beijing.

[63] Nakano Y., Asada K. (1981). Hydrogen peroxide is scavenged by ascorbate-specific peroxidase in spinach chloroplasts. Plant Cell Physiol..

[64] Tagele S.B., Kim S.W., Lee H.G., Lee Y.S. (2019). Aggressiveness and fumonisins production of *Fusarium Subglutinans* and *Fusarium Temperatum* on Korean maize cultivars. Agronomy.

[65] Mayer A.M., Harel E., Ben-Shaul R. (1966). Assay of catechol oxidase—A critical comparison of methods. Phytochemistry.

[66] Singleton V.L., Rossi J.A. (1965). Colorimetry of total phenolics with phosphomolybdic-phosphotungstic acid reagents. Am. J. Enol. Vitic..

[67] Adnan M., Azad M.O.K., Ju H.S., Son J.M., Park C.H., Shin M.H., Alle M., Cho D.H. (2019). Development of biopolymer-mediated nanocomposites using hot-melt extrusion to enhance the bio-accessibility and antioxidant capacity of kenaf seed flour. Appl. Nanosci..

[68] Braca A., Fico G., Morelli I., De Simone F., Tomè F., De Tommasi N. (2003). Antioxidant and free radical scavenging activity of flavonol glycosides from different *Aconitum* species. J. Ethnopharmacol..

[69] Shaw B., Thomas T.H., Cooke D.T. (2002). Responses of sugar beet (*Beta vulgaris* L.) to drought and nutrient deficiency stress. Plant Growth Regul..

[70] Pilon-Smits E.A.H., Terry N., Sears T., van Dun K. (1999). Enhanced drought resistance in fructan-producing sugar beet. Plant Physiol. Biochem..

[71] Ribaut J.-M., Betran J., Monneveux P., Setter T. (2009). Drought tolerance in maize. Handbook of Maize: Its Biology.

[72] Lobell D.B., Roberts M.J., Schlenker W., Braun N., Little B.B., Rejesus R.M., Hammer G.L. (2014). Greater sensitivity to drought accompanies maize yield increase in the US Midwest. Science.

[73] Mou B., He B.-J., Zhao D.-X., Chau K. (2017). Numerical simulation of the effects of building dimensional variation on wind pressure distribution. Eng. Appl. Comput. Fluid Mech..

[74] Efeoğlu B., Ekmekçi Y., Çiçek N. (2009). Physiological responses of three maize cultivars to drought stress and recovery. South Afr. J. Bot..

[75] Hosseini S.A., Réthoré E., Pluchon S., Ali N., Billiot B., Yvin J.-C. (2019). Calcium application enhances drought stress tolerance in sugar beet and promotes plant biomass and beetroot sucrose concentration. Int. J. Mol. Sci..

[76] Ashraf M., Harris P.J.C. (2013). Photosynthesis under stressful environments: An overview. Photosynthetica.

[77] Mibei E.K., Ambuko J., Giovannoni J.J., Onyango A.N., Owino W.O. (2017). Carotenoid profiling of the leaves of selected African eggplant accessions subjected to drought stress. Food Sci. Nutr..

[78] Kaewsuksaeng S. (2011). Chlorophyll degradation in horticultural crops. Walailak J. Sci. Technol..

[79] Ekmekci Y., Bohms A., Thomson J.A., Mundree S.G. (2005). Photochemical and antioxidant responses in the leaves of *Xerophyta viscosa* Baker and *Digitaria sanguinalis* L. under water deficit. Z. Nat. C.

[80] Souza R.P., Machado E.C., Silva J.A.B., Lagôa A., Silveira J.A.G. (2004). Photosynthetic gas exchange, chlorophyll fluorescence and some associated metabolic changes in cowpea (*Vigna unguiculata*) during water stress and recovery. Environ. Exp. Bot..

[81] Akhkha A. (2016). The effect of water stress on photosynthesis, respiration and relative chlorophyll index of the desert plant *Calotropis procera*. Biosci. Biotechnol. Res. Asia.

[82] Hasheminasab H., Aliakbari A., Baniasadi R. (2014). Optimizing the relative water protection (RWP) as novel approach for monitoring drought tolerance in Iranian pistachio cultivars using graphical analysis. Int. J. Biosci..

[83] Min Z., Li R., Chen L., Zhang Y., Li Z., Liu M., Ju Y., Fang Y. (2019). Alleviation of drought stress in grapevine by foliar-applied strigolactones. Plant Physiol. Biochem..

[84] Van Ha C., Leyva-González M.A., Osakabe Y., Tran U.T., Nishiyama R., Watanabe Y., Tanaka M., Seki M., Yamaguchi S., Van Dong N. (2014). Positive regulatory role of strigolactone in plant responses to drought and salt stress. Proc. Natl. Acad. Sci. USA.

[85] Yeilaghi H., Arzani A., Ghaderian M., Fotovat R., Feizi M., Pourdad S.S. (2012). Effect of salinity on seed oil content and fatty acid composition of safflower (*Carthamus tinctorius* L.) genotypes. Food Chem..

[86] Ghaffari H., Tadayon M.R., Nadeem M., Cheema M., Razmjoo J. (2019). Proline-mediated changes in antioxidant enzymatic activities and the physiology of sugar beet under drought stress. Acta Physiol. Plant..

[87] Al-Jbawi E., Abbas F. (2013). The effect of length during drought stress on sugar beet (*Beta vulgaris* L.) yield and quality. Persian Gulf Crop Prot..

[88] Lin C.C., Kao C.H. (2000). Effect of NaCl stress on H_2_O_2_ metabolism in rice leaves. Plant Growth Regul..

[89] Hernández J.A., Almansa M.S. (2002). Short-term effects of salt stress on antioxidant systems and leaf water relations of pea leaves. Physiol. Plant..

[90] Scandalios J.G. (1993). Oxygen stress and superoxide dismutases. Plant Physiol..

[91] Borišev M., Borišev I., Župunski M., Arsenov D., Pajević S., Ćurčić Ž., Vasin J., Djordjevic A. (2016). Drought impact is alleviated in sugar beets (*Beta vulgaris* L.) by foliar application of fullerenol nanoparticles. PLoS ONE.

[92] Zhao L., Peng B., Hernandez-Viezcas J.A., Rico C., Sun Y., Peralta-Videa J.R., Tang X., Niu G., Jin L., Varela-Ramirez A. (2012). Stress response and tolerance of *Zea mays* to CeO_2_ nanoparticles: Cross talk among H_2_O_2_, heat shock protein, and lipid peroxidation. ACS Nano.

[93] Sarker U., Oba S. (2018). Drought stress effects on growth, ROS markers, compatible solutes, phenolics, flavonoids, and antioxidant activity in *Amaranthus tricolor*. Appl. Biochem. Biotechnol..

[94] Putnik-Delić M.I., Maksimović I.V., Nikolić-Đorić E.B., Nagl N.M. (2010). Analyses of statistical transformations of row data describing free proline concentration in sugar beet exposed to drought. Zb. Matice Srp. Prir. Nauk..

[95] Szabados L., Savoure A. (2010). Proline: A multifunctional amino acid. Trends Plant Sci..

[96] Hong-Bo S., Xiao-Yan C., Li-Ye C., Xi-Ning Z., Gang W., Yong-Bing Y., Chang-Xing Z., Zan-Min H. (2006). Investigation on the relationship of proline with wheat anti-drought under soil water deficits. Colloids Surf. B Biointerfaces.

[97] Farooq A., Bukhari S.A., Akram N.A., Ashraf M., Wijaya L., Alyemeni M.N., Ahmad P. (2020). Exogenously applied ascorbic acid-mediated changes in osmoprotection and oxidative defense system enhanced water stress tolerance in different cultivars of safflower (*Carthamus tinctorious* L.). Plants.

[98] Ashraf M., Foolad M.R. (2007). Roles of glycine betaine and proline in improving plant abiotic stress resistance. Environ. Exp. Bot..

[99] Anjum S.A., Wang L.C., Farooq M., Hussain M., Xue L.L., Zou C.M. (2011). Brassinolide application improves the drought tolerance in maize through modulation of enzymatic antioxidants and leaf gas exchange. J. Agron. Crop Sci..

[100] Genard H., Le Saos J., Billard J.-P., Tremolieres A., Boucaud J. (1991). Effect of salinity on lipid composition, glycine betaine content and photosynthetic activity in chloroplasts of *Suaeda maritima*. Plant Physiol. Biochem..

[101] Giri J. (2011). Glycinebetaine and abiotic stress tolerance in plants. Plant Signal. Behav..

[102] Hoekstra F.A., Golovina E.A., Buitink J. (2001). Mechanisms of plant desiccation tolerance. Trends Plant Sci..

[103] Karimi S., Yadollahi A., Arzani K. (2013). Responses of almond genotypes to osmotic stress induced in vitro. J. Nuts.

[104] Ashraf M. (2009). Biotechnological approach of improving plant salt tolerance using antioxidants as markers. Biotechnol. Adv..

[105] Wang W.-B., Kim Y.-H., Lee H.-S., Kim K.-Y., Deng X.-P., Kwak S.-S. (2009). Analysis of antioxidant enzyme activity during germination of alfalfa under salt and drought stresses. Plant Physiol. Biochem..

[106] Alscher R.G., Erturk N., Heath L.S. (2002). Role of superoxide dismutases (SODs) in controlling oxidative stress in plants. J. Exp. Bot..

[107] Jaleel C.A., Riadh K., Gopi R., Manivannan P., Inès J., Al-Juburi H.J., Chang-Xing Z., Hong-Bo S., Panneerselvam R. (2009). Antioxidant defense responses: Physiological plasticity in higher plants under abiotic constraints. Acta Physiol. Plant..

[108] DaCosta M., Huang B. (2007). Changes in antioxidant enzyme activities and lipid peroxidation for bentgrass species in response to drought stress. J. Am. Soc. Hortic. Sci..

[109] Almeselmani M., Deshmukh P.S., Sairam R.K., Kushwaha S.R., Singh T.P. (2006). Protective role of antioxidant enzymes under high temperature stress. Plant Sci..

[110] Shen C., Zhang Q., Li J., Bi F., Yao N. (2010). Induction of programmed cell death in *Arabidopsis* and rice by single-wall carbon nanotubes. Am. J. Bot..

[111] Gill S.S., Tuteja N. (2010). Reactive oxygen species and antioxidant machinery in abiotic stress tolerance in crop plants. Plant Physiol. Biochem..

[112] Sekmen A.H., Ozgur R., Uzilday B., Turkan I. (2014). Reactive oxygen species scavenging capacities of cotton (*Gossypium hirsutum*) cultivars under combined drought and heat induced oxidative stress. Environ. Exp. Bot..

[113] Alam M.M., Nahar K., Hasanuzzaman M., Fujita M. (2014). Exogenous jasmonic acid modulates the physiology, antioxidant defense and glyoxalase systems in imparting drought stress tolerance in different *Brassica* species. Plant Biotechnol. Rep..

[114] Boeckx T., Winters A.L., Webb K.J., Kingston-Smith A.H. (2015). Polyphenol oxidase in leaves: Is there any significance to the chloroplastic localization?. J. Exp. Bot..

[115] Lee B.-R., Kim K.-Y., Jung W.-J., Avice J.-C., Ourry A., Kim T.-H. (2007). Peroxidases and lignification in relation to the intensity of water-deficit stress in white clover (*Trifolium repens* L.). J. Exp. Bot..

[116] Thipyapong P., Melkonian J., Wolfe D.W., Steffens J.C. (2004). Suppression of polyphenol oxidases increases stress tolerance in tomato. Plant Sci..

[117] Mayer A.M., Harel E. (1979). Polyphenol oxidases in plants. Phytochemistry.

[118] Vaughn K.C., Duke S.O. (1984). Function of polyphenol oxidase in higher plants. Physiol. Plant..

[119] Halliwell B. (1975). Hydroxylation of p-Coumaric acid by illuminated chloroplasts. The role of superoxide. Eur. J. Biochem..

[120] Steffens J.C., Harel E., Hunt M.D. (1994). Polyphenol oxidase. Genetic Engineering of Plant Secondary Metabolism.

[121] Sairam R.K., Srivastava G.C., Saxena D.C. (2000). Increased antioxidant activity under elevated temperatures: A mechanism of heat stress tolerance in wheat genotypes. Biol. Plant..

[122] Sarker U., Oba S. (2018). Drought stress enhances nutritional and bioactive compounds, phenolic acids and antioxidant capacity of *Amaranthus* leafy vegetable. BMC Plant Biol..

[123] Bartwal A., Mall R., Lohani P., Guru S.K., Arora S. (2013). Role of secondary metabolites and brassinosteroids in plant defense against environmental stresses. J. Plant Growth Regul..

[124] Gharibi S., Tabatabaei B.E.S., Saeidi G., Goli S.A.H. (2016). Effect of drought stress on total phenolic, lipid peroxidation, and antioxidant activity of *Achillea* species. Appl. Biochem. Biotechnol..

[125] Easwar Rao D., Viswanatha Chaitanya K. (2019). Changes in the antioxidant intensities of seven different soybean (*Glycine max* (L.) Merr.) cultivars during drought. J. Food Biochem..

[126] Espinoza A., San Martín A., López-Climent M., Ruiz-Lara S., Gómez-Cadenas A., Casaretto J.A. (2013). Engineered drought-induced biosynthesis of α-tocopherol alleviates stress-induced leaf damage in tobacco. J. Plant Physiol..

[127] Bettaieb I., Hamrouni-Sellami I., Bourgou S., Limam F., Marzouk B. (2011). Drought effects on polyphenol composition and antioxidant activities in aerial parts of *Salvia officinalis* L.. Acta Physiol. Plant..

[128] Lin K.-H., Chao P.-Y., Yang C.-M., Cheng W.-C., Lo H.-F., Chang T.-R. (2006). The effects of flooding and drought stresses on the antioxidant constituents in sweet potato leaves. Bot. Stud..

